# Ferrous and Ferric Ion-Facilitated Dilute Acid Pretreatment of Lignocellulosic Biomass under Anaerobic or Aerobic Conditions: Observations of Fe Valence Interchange and the Role of Fenton Reaction

**DOI:** 10.3390/molecules25061427

**Published:** 2020-03-20

**Authors:** Hui Wei, Wei Wang, Peter N. Ciesielski, Bryon S. Donohoe, Min Zhang, Michael E. Himmel, Xiaowen Chen, Melvin P. Tucker

**Affiliations:** 1 Biosciences Center, National Renewable Energy Laboratory, Golden, CO 80401, USA; Wei.Wang@nrel.gov (W.W.); Peter.Ciesielski@nrel.gov (P.N.C.); bryon.donohoe@nrel.gov (B.S.D.); Min.Zhang@nrel.gov (M.Z.); mike.himmel@nrel.gov (M.E.H.); 2 National Bioenergy Center, National Renewable Energy Laboratory, Golden, CO 80401, USA

**Keywords:** dilute acid pretreatment, metal co-catalyst, ferric ions, ferrous ions, biomass, cellulose, corn stover, hydrogen peroxide, Fenton reaction

## Abstract

Ferrous ion co-catalyst enhancement of dilute-acid (DA) pretreatment of biomass is a promising technology for increasing the release of sugars from recalcitrant lignocellulosic biomass. However, due to the reductive status of ferrous ion and its susceptibility to oxidation with exposure to atmosphere, its effective application presumably requires anaerobic aqueous conditions created by nitrogen gas-purging, which adds extra costs. The objective of this study was to assess the effectiveness of oxidative iron ion, (i.e., ferric ion) as a co-catalyst in DA pretreatment of biomass, using an anaerobic chamber to strictly control exposure to oxygen during setup and post-pretreatment analyses. Remarkably, the ferric ions were found to be as efficient as ferrous ions in enhancing sugar release during DA pretreatment of biomass, which may be attributed to the observation that a major portion of the initial ferric ions were converted to ferrous during pretreatment. Furthermore, the detection of hydrogen peroxide in the liquors after DA/Fe ion pretreatment suggests that Fenton reaction chemistry was likely involved in DA/Fe ion pretreatments of biomass, contributing to the observed ferric and ferrous interchanges during pretreatment. These results help define the extent and specification requirements for applying iron ions as co-catalysts in DA pretreatments of biomass.

## 1. Introduction

Lignocellulosic biomass is largely composed of three major constituents: cellulose, hemicellulose and lignin [1]. The utilization of cellulose and hemicellulose for the production of biofuels has been widely studied [2,3,4,5,6]. However, the efficient conversion of lignocellulosic biomass into fermentable monosaccharides still remains a difficult task due to the recalcitrance of biomass to deconstruction [7].

As one of the leading biomass conversion technologies, pretreatment of lignocellulosic biomass with dilute acid (DA) at mild conditions effectively liberates hemicellulose, modifies lignin networks, and increases cellulose accessibility [8,9]. However, DA pretreatment presents several challenges as well as opportunities for optimization, including improving sulfuric acid recovery, lowering the need for neutralization of pretreated biomass prior to enzymatic saccharification, and reducing the need for acid resistant reactors with expensive metallurgy. Additionally, precise process control such as adjusting the pretreatment severity is required to minimize the potential degradation of desired monomeric sugars into dehydration products that inhibit the downstream fermentation. Encouragingly, these challenges can be mitigated through integrating accessory processes such as deacetylation [10] or adding a co-catalyst into DA pretreatment, as described below.

As a co-catalyst, ferrous ion (hereafter referred as Fe^2+^) was found to enhance DA pretreatment of biomass, increasing the release of sugars during pretreatment or subsequent saccharification [11]. Nguyen and Tucker reported a 20%–30% improvement in overall sugar yield released from softwood by incorporating 0.5–2.0 mM ferrous sulfate during the DA impregnation stage [11]. The economic benefit of adding Fe^2+^ ions can be attributed to lowering the severity of pretreatment conditions (a composite factor based on temperature, acid concentration and time), while maintaining comparable conversion to biomass sugars during pretreatment and cellulose digestibility during enzymatic hydrolysis. For the downstream fermentation of DA-generated hydrolysates, ferrous sulfate coupled with hydrogen peroxide has recently been used to detoxify hydrolysates of spruce wood, improving fermentation yields [12].

Advances have been made in understanding the mechanism of Fe^2+^-facilitated DA pretreatment of biomass. Fe^2+^ co-catalyst has been shown to catalyze xylan hydrolysis and improve xylose yields during pretreatment compared to DA pretreatment alone at the same conditions [11]. Our group’s recent study revealed that DA/Fe^2+^ ion pretreatment enhances solubilization and enzymatic digestion of both cellulose and xylan to simple sugars. DA/Fe^2+^ ion pretreatment targets multiple chemistries, including the C-O-C and C-H bonds of cellulose, in plant cell wall polymer networks [13]. Fe^2+^ ions were found to be associated with both cellulose/xylan and lignin in pretreated corn stover samples [13]. Furthermore, a recent study demonstrated that iron ion catalyst increases the selectivity of acid hydrolysis of amorphous over crystalline regions of cellulose under augmented hydrolysis conditions [14].

Nevertheless, there is an inherent shortcoming in using Fe^2+^ ion as a co-catalyst due to its susceptibility to being oxidized to Fe^3+^ ions. Thus, the current Fe^2+^ ion facilitated DA pretreatment had been conducted in an aqueous liquid purged with N_2_ gas [11], limiting its practical implementation. To circumvent this restraint, recent tests of DA/Fe^3+^ pretreatment of corn stover showed its effectiveness in increasing the overall enzymatic digestibility of biomass residues [15]. However, despite the above studies, we still lack a fundamental understanding of the underlying mechanism for the effectiveness of both DA/Fe^2+^ and DA/Fe^3+^ pretreatments. One question is if the Fenton reaction is involved in DA/Fe ion pretreatment process, as it partially meets the condition of Fenton reaction in terms of Fe ion as catalyst and the acidic pH solvent.

The objectives of this study are first to assess the effectiveness of ferric versus ferrous ions as a co-catalyst in DA pretreatment of lignocellulosic biomass, second to determine the fate of Fe^3+^ ion during the pretreatment under aerobic and anaerobic conditions, and finally to explore the possibility that the Fenton reaction is part of the mechanisms underlying metal ion-facilitated DA pretreatment. The insights gained will be crucial for defining the extent and specification for applying iron ion co-catalysts in DA pretreatments of biomass.

## 2. Results and Discussion

### 2.1. TEM Imaging of Dilute Acid/Fe^3+^ Ions-Pretreated Corn Stover under Anaerobic Setup Condition

**Figure 1:**
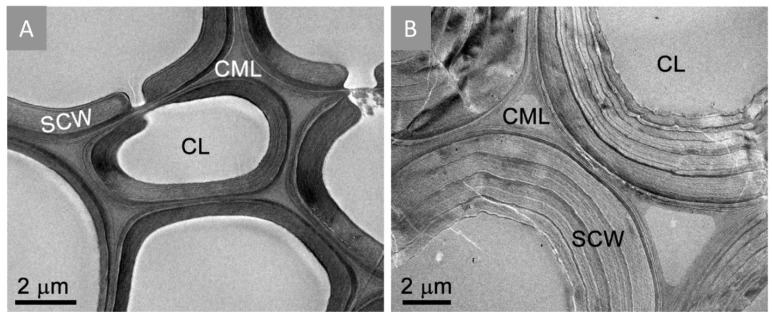
Transmission electron micrographs of native (**A**) and pretreated (**B**) corn stover cell walls. Pretreatment removed structural hemicelluloses and relocated lignin, which allowed for loosening and expansion of the cell wall. Annotations: SCW, secondary cell wall; CL, cell lumen; CML, compound middle lamella.

Previously, we employed transmission electron microscopy (TEM) and scanning electron microscopy (SEM) to study the effects of anaerobic DA/Fe^2+^ ion pretreatment on the ultrastructural of biomass residues. The results reveal the occurrence of delamination and fibrillation to the cell wall of biomass after DA/Fe^3+^ ion pretreatment [13]. In this study, we extended the TEM analysis to visualize ultrastructural modifications to the cell wall resulting from the anaerobic DA/Fe^3+^ ion pretreatment process. Vascular tissue was selected for structural examination to investigate the impact of the pretreatment on the heavily lignified and recalcitrant secondary cell walls that are typical of this tissue type. The untreated cell walls are shown in Figure 1A and pretreated cell walls are shown in Figure 1B. The pretreated cell walls exhibit swelling, which results from a general loosening of the lignocellulosic matrix due the removal of hemicelluloses and relocation of lignin [16]. It is noteworthy that previous structural investigations of cell walls treated with DA with iron sulfate co-catalyst carried out in a steam explosion reactor resulted in extensive nanofibrillation of the cell wall [17], which was not observed in the present samples pretreated in 65-mL pressure tubes using the Parr reactor.

### 2.2. Efficiency of DA/Fe Pretreatment under Anaerobic Condition: Fe^3+^ Is Nearly as Effective as Fe^2+^ in Enhancing DA Pretreatment of Biomass

Previous studies have shown that the effective concentration range for Fe^2+^ enhancing the efficiency of DA pretreatment of biomass is 1–10 mM. To facilitate the measurement of Fe in each fraction of pretreated biomass mixture, 5–10 mM Fe^2+^ or Fe^3+^ were chosen for the DA pretreatment of corn stover. The results demonstrate that DA/5–10 mM Fe^2+^ and Fe^3+^ are both efficient in releasing more glucose and xylose than DA pretreatment alone under anaerobic condition (Table 1). It is intriguing that Fe^3+^ is nearly as effective as Fe^2+^ in enhancing DA pretreatment (Table 1, Rows 4–5 versus 2–3).

### 2.3. Fe Ion Interconversion and Specification in Hydrolysates After DA/Fe Pretreatment Under Anaerobic Condition

**Figure 2:**
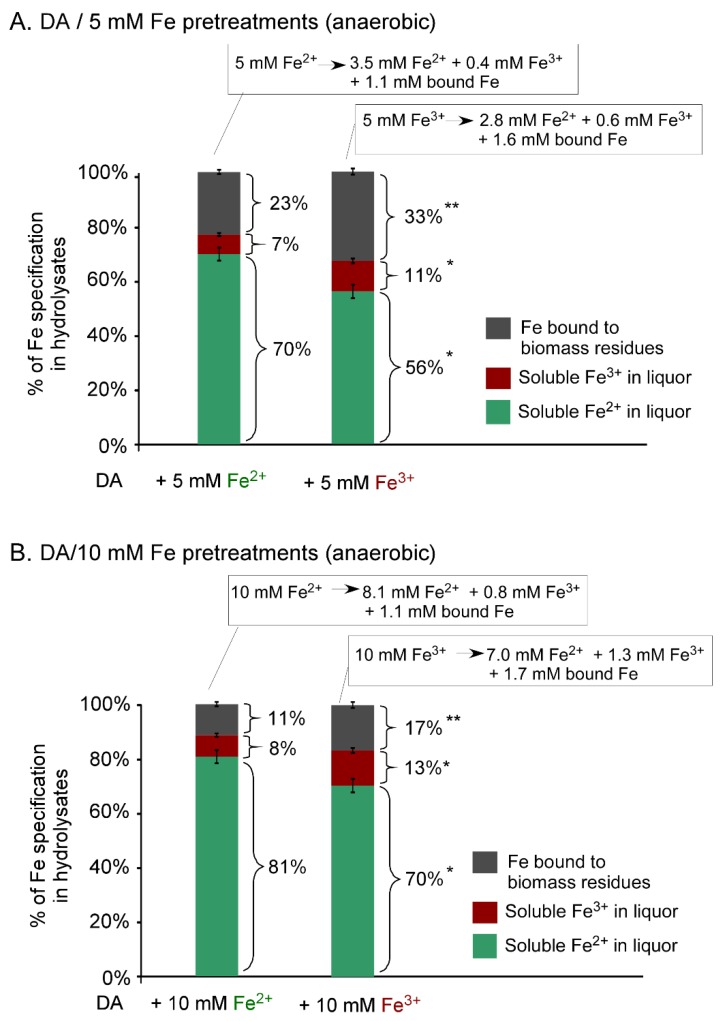
Fate of Fe in corn stover pretreated with either 5 or 10 mM Fe ions in dilute acid (0.5% H_2_SO_4_) at 150 °C for 20 min under anaerobic conditions. (**A**) Pretreatments of CS with DA/5 mM Fe^2+^ or Fe^3+^ ions. (**B**) Pretreatments of CS with DA/10 mM Fe^2+^ or Fe^3+^ ions. Soluble Fe^2+^ or Fe^3+^ were measured in the liquor after biomass pretreatment. The colors used in the bars: green for Fe^2+^ and red for Fe^3+^ in liquor; black for Fe bound to biomass residues. While the bars represent the percentage of Fe specification in the hydrolysates, the equations in each associated insert represent the valence and concentration of soluble Fe ions before and after the pretreatments. Asterisk indicates statistically significant difference between Fe^2+^ and Fe^3+^ pretreatments (* for *p* < 0.05; ** for *p* < 0.01). CS, corn stover; DA, dilute acid.

As Fe is a transition metal element that can undergo active conversion between ferrous and ferric states, we hypothesized that the Fe^3+^ ions in DA/Fe^3+^ pretreatment was converted to Fe^2+^ during the pretreatment process. To test this hypothesis, we scaled up the DA/Fe^2+^ and DA/Fe^3+^ pretreatment of corn stover (3 g biomass/30 mL solvent) in pressure tubes. The scale-up pretreatment generated enough pretreated biomass solids to be nitric acid-digested and allowed the measurement of Fe in the solid biomass fraction, while the concentration of soluble Fe^2+^ and Fe^3+^ ions was measured in the liquor fraction. The soluble Fe ion concentrations in the liquor are shown in the inserted equations, while the percentage of Fe specification in the liquor versus solid biomass fractions is presented in Figure 2.

After the pretreatment of corn stover with DA/5 mM Fe^3+^ ions, surprisingly, 2.8 mM Fe^2+^ ions were found in the liquor, indicated that 56% of the initial added Fe^3+^ was reduced to Fe^2+^ during the pretreatment (Figure 2A, right bar and the linked equation); the overall Fe specification distribution was 56% (i.e., 2.8 mM), 11% (i.e., 0.6 mM) and 33% (1.6 mM) of the iron was found in the forms of soluble Fe^2+^, soluble Fe^3+^ and bound Fe, respectively, in the hydrolysates after pretreatment (Figure 2A, right bar). Similarly, after DA/10 mM Fe^3+^ pretreatment of corn stover, 70% (i.e., 7.0 mM), 13% (i.e., 1.3 mM) and 17% (i.e., 1.7 mM) of the iron was in the forms of soluble Fe^2+^, soluble Fe^3+^ and bound Fe in the hydrolysate volume, respectively (Figure 2B, right bar). Thus, in both DA/5 mM and 10 mM Fe^3+^ pretreatments, over half of the Fe was in the form of Fe^2+^, supporting our hypothesis that the Fe^3+^ ions in DA/Fe^3+^ pretreatment was converted to Fe^2+^ during the pretreatment process.

In the case of anaerobic DA/5 mM and 10 mM Fe^2+^ pretreatment of corn stover, a similar Fe specification distribution was observed but with an understandably relatively higher portion of Fe^2+^ form, attributed to the fact that Fe^2+^ is the form of iron initially added into the DA and started with. Specifically, after DA/Fe^2+^ pretreatment, 70% to 81%, 7% to 8%, and 23% to 11% of the iron was found to exist in the forms of soluble Fe^2+^, soluble Fe^3+^ and bound Fe in the volume of hydrolysate, respectively (Figure 2A,B, left bars).

### 2.4. Impacts of Oxygen on the Pretreatment Efficiency and the Fe Valence Changes

To assess the possibility of applying the DA/Fe pretreatment of biomass under aerobic conditions, we also conducted the DA/Fe^2+^ and DA/Fe^3+^ pretreatments of corn stover with the solvent (i.e., DA) saturated with oxygen instead of being sparged with nitrogen gas.

#### 2.4.1. Pretreatment Efficiency under Aerobic Conditions

The sugar release data revealed that under aerobic conditions, both 5 and 10 mM FeCl_2_ and FeCl_3_ still increase the glucose and xylose release compared to DA alone pretreatment (Table 2), but the magnitude of the enhancement is lower compared to the anaerobic conditions where oxygen was completely expelled with N_2_-purging (see Table 2 versus Table 1). Overall, the data indicate that the presence of oxygen in the pretreatment solvent only slightly compromised the efficacy of 5 to 10 mM Fe^2+^ and Fe^3+^ in enhancing DA pretreatment.

#### 2.4.2. Fe^3+^ to Fe^2+^ Conversion under Aerobic Conditions

To determine if and to what extent oxygen affects the conversion of Fe^3+^ ions to Fe^2+^ ions during aerobic DA/5 mM Fe^3+^ pretreatment, the Fe^2+^ concentration in the liquor was measured. The results show that after aerobic DA/5 mM Fe^3+^ pretreatment, its Fe valence conversion (5 mM Fe^3+^ → 1.9 mM Fe^2+^, i.e., 38% of the Fe^3+^) was lower than that after anaerobic DA/5 mM Fe3+ pretreatment (5 mM Fe^3+^ → 2.8 mM Fe^2+^; as observed in earlier section illustrated in Figure 2A, the eqn linked to right bar). Nevertheless, although less Fe^2+^ was formed from Fe^3+^ under aerobic conditions, the presence of 1.9 mM [Fe^2+^] ions is still substantial, and partially accounts for the observed efficacy of DA/Fe^3+^ pretreatment under aerobic conditions (Table 2). Overall, the above observation of Fe valence changes under both aerobic and anaerobic conditions prompted us to explore the possible mechanism behind them.

### 2.5. Detection of Hydrogen Peroxide

**Figure 3:**
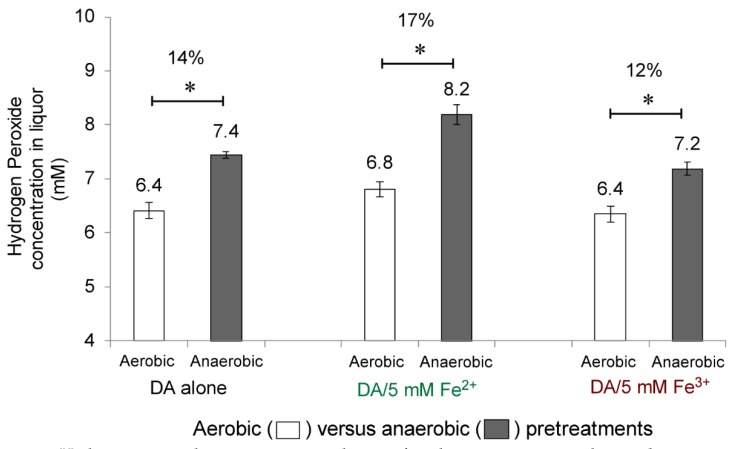
Hydrogen peroxide concentration in liquor after the pretreatment under aerobic versus anaerobic conditions. Asterisk indicates statistically significant difference between the compared two groups (* for *p* < 0.05). DA, dilute acid.

To explore the mechanism of the observed Fe^2+^ and Fe^3+^ interchanges under both aerobic and anaerobic DA/Fe pretreatments of biomass, we hypothesized that a thermal Fenton reaction is involved. To test this hypothesis, we measured the concentration of hydrogen peroxide in the liquor after the DA/Fe pretreatment of corn stover. Concentrations of 6 to 8 mM hydrogen peroxide were detected in all the liquor samples generated from DA alone, DA/Fe^2+^ and DA/Fe^3+^ pretreatments, under both aerobic and anaerobic condition (Figure 3). Specifically, DA alone, DA/5 mM Fe^2+^, and DA/5 mM Fe^3+^ generated 14%, 17% and 12% more hydrogen peroxide under the anaerobic condition than under the aerobic condition, respectively (Figure 3, filled versus open bars). The higher concentration of hydrogen peroxide generated under anaerobic versus aerobic conditions may have contributed to the observed higher efficiency in simple sugar release under anaerobic versus aerobic conditions (Table 1 versus Table 2).

### 2.6. A Possible Role of Thermal Fenton Reaction in DA/Fe Pretreatments

The detection of in situ generated hydrogen peroxide in the liquor of DA/Fe ion pretreatment supports our proposal of Fenton reaction involvement in the DA/Fe pretreatment process, which can explain the observed Fe^2+^ and Fe^3+^ interchanged by the DA/Fe ion pretreatments.

The Fenton reaction was first described in 1894 [18], and the conventional Fenton reactions in which hydrogen peroxide and Fe ions are defined as Fenton’s reagents are illustrated in Equations (1) and (2). The core part of Fenton process is the catalytic cycle of the reactions between Fe ions (catalyst) and hydrogen peroxide (oxidant) to produce highly reactive hydroxyl radical (OH•) under the acidic pH condition [19,20]:

Fe^2+^ + H_2_O_2_ → Fe^3+^ + OH^−^ + OH* (1)

Fe^3+^ + H_2_O_2_ → Fe^2+^ + OOH^−^ + H^+^ (2)

The chemical reactions illustrated in Equations (1) and (2) can mediate the recycling of Fe^2+^ and Fe^3+^ during DA/Fe pretreatment.

While some previous studies have suggested that Fe^2+^ ions may be preferred when low doses of Fenton’s reagent are used (e.g., < 0.3–0.7 mM H_2_O_2_, and/or < 1 mM Fe ions) [21,22], others showed that it did not matter much whether Fe^2+^ or Fe^3+^ ions are utilized to catalyze the reaction for applications like degrading environmental pollutants such as chlorophenols [22]. Here, our results support that both Fe^2+^ and Fe^3+^ ions were effective in breaking down biomass to simple sugars by DA/Fe ion pretreatments. DA/Fe ion pretreatments can be viewed as a type of thermal Fenton reaction, during which the cycling between Fe^2+^ and Fe^3+^ allows the Fenton reaction to generate hydroxyl radicals that can contribute to the breakdown and conversion of biomass to simple sugars.

### 2.7. Future Studies on the Effect of IRON Facilitated DA Pretreatment on Components of Biomass

The results of this study were derived from the pretreatment of CS, which was processed from maize, a monocot and a member of the grass family. Since corn stover can vary based on the location and time it was planted and harvested, the natural variation of this biomass feedstock may have had an impact on the results that were observed. Future studies are warranted to investigate the effect of these reactions on model feedstocks such as extracted cellulose or xylan and the effects this would have had on the resulting Fe^2+^ and Fe^3+^ in the aerobic and anaerobic conditions. These studies may provide further insights into the mechanisms on different components (cellulose, xylose and lignin) of biomass involved in iron facilitated dilute acid pretreatment of biomass.

## 3. Materials and Methods

### 3.1. Main Experimental Steps andAapparatus

**Figure 4:**
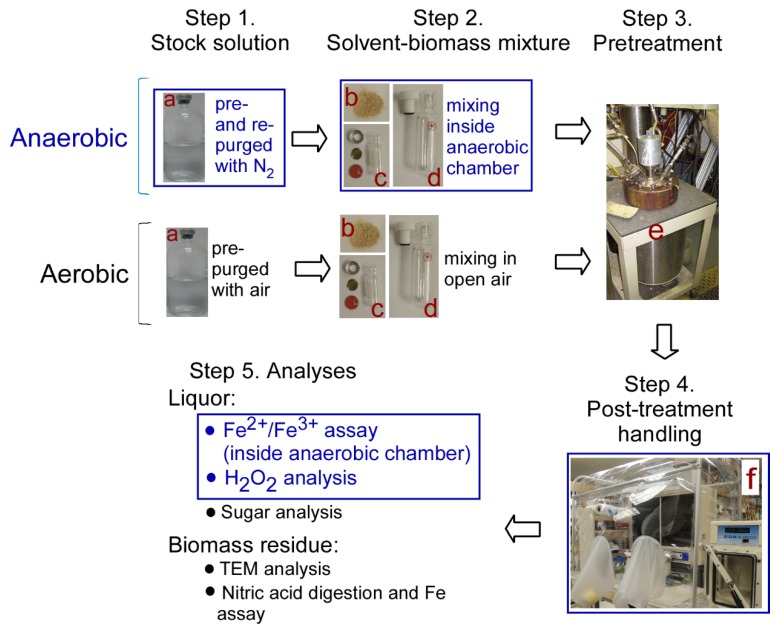
Flow-chart of the main steps and apparatus in anaerobic versus aerobic pretreatments, subsequent handling and analytic processes. Blue outlines indicate the related experiments conducted with N_2_ purging or inside an anaerobic chamber. Materials and apparatus used: **a**: 250-mL serum bottle; **b**: corn stover; **c**: 2-mL glass vials with copper disc and septum/aluminum cap for regular scale pretreatment; **d**: 65-mL pressure tubes for scale-up pretreatment; **e**: Parr reactor; **f**: Anaerobic chamber. Abbreviations: HPLC, high-performance liquid chromatography; TEM, transmission electron microscopy.

The overall scheme for the five-step experimental approach is illustrated in Figure 4. The first two steps were different for the aerobic versus anaerobic setups in preparing the stock solution and the biomass–solvent mixtures, while the remaining steps were common to both experiments and are described in detail in the following sections.

#### 3.1.1. Step 1. Solvent Stock Solution

The main chemicals used were 96% sulfuric acid (catalog no. 9681–03; J. T. Baker Chemical Co., Phillipsburg, NJ, USA), FeCl_2_·4H_2_O (catalog no. 44939; Sigma-Aldrich, St. Louis, MO, USA), and FeCl_3_·6H_2_O (catalog no. 236480; Sigma-Aldrich, St. Louis, MO, USA). For DA/metal ion co-catalyst pretreatment, the 0.5 wt% H_2_SO_4_ was prepared beforehand. One hundred milliliters of acid solution was distributed into a 250-mL serum bottle, and the solvent was pre-sparged for 5 min with either air to make it oxygen-saturated (for aerobic setup condition), or N_2_ gas (for anaerobic setup condition) using a long needle that reached to the bottom of the bottle. Then, an appropriate amount of FeCl_2_ or FeCl_3_ was added into the bottle to reach the designated concentrations. Following that, the dilute sulfuric acid/metal ion co-catalyst solution was directly capped (for aerobic setup condition), or re-sparged with N_2_ gas for 5 min (for anaerobic setup condition), and then the bottles were capped with Teflon stoppers and aluminum crimps (Figure 4, step 1).

#### 3.1.2. Step 2. Biomass–Solvent Mixtures

Biomass–solvent mixtures, as illustrated in Figure 4, step 2, were prepared either in the open air for pretreatment designated with aerobic setup condition, or inside an anaerobic chamber (Coy Laboratory Products Inc., Grass Lake, MI, USA) with a standard mix of 5% hydrogen gas and 95% nitrogen gas, which is classified as non-combustible for pretreatment designated with anaerobic setup condition. The biomass corn stover (CS) used is described in the notes of Table 3. The containers used were either 2-mL HPLC glass vials with septum/aluminum caps (item c in Figure 4) for regular scale pretreatment, or 65-mL pressure tubes with o-ring seals and screw-on Teflon caps (Ace glass # 8648–30 tube with #5845–47 plug; Ace Glass Inc, Vineland, NJ, USA; as illustrated in item d in Figure 4) for scale-up pretreatment.

The detailed makeup the biomass–solvent mixtures is listed in Table 3. In brief, the regular scale pretreatment contained 100 mg biomass in 1 mL solvent (DA), which led to 10% (*w*/*v*) biomass loading. In contrast, the scale-up pretreatment contained 3 g milled corn stover powder in 30 mL solvent.

After sealing the glass container, the biomass–solvent mixtures were allowed to settle overnight for solvent diffusion and impregnation of biomass, followed by pretreatment in the following day.

#### 3.1.3. Step 3. Pretreatment Conditions

The above sealed 2 mL glass vials and 65 mL pressure tubes were contained in a metal wire-made net and lowered into a two-gallon Parr reactor (Figure 4, step 3; Parr Instrument Co., Moline, IL, USA). The glass vials and pressure tubes were then rapidly heated to the target temperature of 150 °C with steam. After 20 min reaction time, the Parr reactor was cooled down to around 50 °C with a flush of cooling water, then the net that holding vials and tubes was removed from the Parr reactor and quenched in ice water to terminate the reaction.

#### 3.1.4. Step 4. Post-Handling of Pretreated Samples

After pretreatment, the vials and tubes were transferred to anaerobic chamber (Figure 4, step 4), where the operation of step 4 mainly was conducted. The pretreated slurry was pipetted out and transferred into 1-mL syringe fitted with a 0.45 µm nylon filter. Ten microliters of the filtrate was used for Fe^2+^ and Fe^3+^ analysis as described in the section below, and the remaining filtrate was analyzed for released sugars using HPLC.

The remaining slurry in the vials and tubes was taken out from the anaerobic chamber, from which the biomass residue pellets were collected by centrifugation at 4000× *g* for 10 min.

### 3.2. Transmission Electron Microscopy

Particles comprising vascular tissue were visually selected from milled native or milled and DA/5 mM Fe^3+^ pretreated corn stover samples under the anaerobic setup condition. The particles were processed via the microwave resin embedding process described previously [23]. Briefly, samples were first infiltrated with water, followed by dehydration with increasing concentrations of ethanol with intermittent microwave steps. Next, the samples were infiltrated with LR White (London Resin Company, Reading, United Kingdom) by incubating at room temperature for several hours (up to overnight) in increasing concentrations of resin diluted in ethanol. Microwave steps were performed each time the concentration was increased. The fully infiltrated samples were capsules and placed in a laboratory oven at 60 °C overnight to polymerize the resin. Ultra-thin (~60 nm) sections were cut using a Leica Ultramicrotome (Leica Microsystems GmbH, Wetzlar, Germany) and collected on 0.5% polyvinyl formvar coated copper slot grids (SPI Supplies, West Chester, PA, USA). Grids were individually stained for 30 s with 1% aqueous KMnO_4_. Images were taken with a four-megapixel Gatan UltraScan 1000 camera (Gatan, Pleasanton, CA, USA) on a FEI Tecnai G2 20 Twin 200 kV LaB6 TEM (FEI, Hillsboro, OR, USA) operated with an accelerating voltage of 200 kV.

### 3.3. HPLC Analysis for the Sugars in Liquors After Pretreatment

The liquors of pretreatment hydrolysates, as prepared in step 4 of the above procedure, were subjected to HPLC analysis for soluble carbohydrates, as described previously [13].

### 3.4. Measurement of Fe^2+^ and Fe^3+^ Ions in Liquors After Pretreatment

The liquors of pretreatment hydrolysates, as prepared in step 3 of the above procedure, were immediately used for the determination of Fe^2+^ and total Fe by QuantiChrom iron assay kit (Bioassay System, Hayward, CA, USA) based on the manufacturer’s instructions. The Fe^3+^ was calculated by the total [Fe]–[Fe^2+^]. The assay was conducted inside an anaerobic chamber (Coy Laboratory Products Inc., Grass Lake, MI, USA).

Briefly, the working reagent for the assay was freshly prepared by mixing the QuantiChrom Reagents (A:B:C at 20:1:1 at volume). Fifty microliters of standard or diluted liquor samples were mixed with 200 µL working reagent in a 96-well plate. After 40 min of incubation at room temperature, the plate was removed from the anaerobic chamber, and the absorption at 590 nm was measured. The iron concentrations were calculated by comparison of the absorbance with the standard curve.

### 3.5. Nitric Acid Digestion of Biomass Residue and the Measurement of Total Fe Ions

The scale-up (i.e., 3 g biomass/30 mL solvent) pretreatment of corn stover generated enough biomass residues that allowed the measurement of Fe in the biomass fraction. The biomass residues were removed from anaerobic chamber to regular bench in open air. The samples were oven dried at 70 °C for 12 h, then digested by nitric acid using a modified procedure based on the literature [24]. Briefly, 200 mg dry biomass residue was ground and digested at 70 °C for overnight in 4 mL 25% (v/v) nitric acid (with Ca^2+^, K^+^ and Mg^2+^ ≤ 0.5 ppb; trace metal grade, Fisher Scientific, Fairlawn, NJ, USA). After digestion, the extract was diluted to 50 mL with fresh Millipore (Synergy water Purification System, Merck KGaA, Darmstadt, Germany) de-ionized H_2_O (the final nitric acid concentration was 2%) and analyzed for the Fe concentration using the above QuantiChrom iron assay kit.

### 3.6. Measurement of Hydrogen Peroxide

High-performance anion exchange chromatography with pulsed amperometric detection (HPAEC-PAD) had been used for the measurement of hydrogen peroxide in the literature [25,26]. The 0.45-µm filtered liquor from DA pretreatments were separated and analyzed using Dionex ICS−3000 HPAEC-PAD system (Thermo Scientific, San Jose, CA, USA) that included a PA−10 column and guard. The flow rate was 1 mL/min, and the mobile phase consisted of 150 mmol sodium acetate. The detector and column were at 40 °C, and the injection volume was 5 µL. The hydrogen peroxide standard was in the range of 0.01–1 g/L.

## 4. Conclusion

Anaerobic conditions improve the ability of both ferrous and ferric ions to enhance sugar release during DA pretreatment of biomass. The ability of Fe^3+^ to enhance the efficiency of DA/Fe^3+^ pretreatment can be at least partially attributed to the conversion of Fe^3+^ to Fe^2+^. The study also revealed that aerobic conditions partially compromise the efficiency of DA/Fe^3+^ and DA/Fe^2+^ pretreatments of biomass, but this compromise can be offset by increasing the concentration of Fe co-catalysts into the range of 5 to 10 mM. This suggests that the application of DA/Fe pretreatments under normal atmospheric condition can still be conducted as an efficient pretreatment technology. The findings help to define the extent and specification requirements for applying iron ion co-catalysts in DA pretreatments at an industrial scale where Fe^3+^ is likely to be more practical than Fe^2+^, and aerobic conditions are more convenient than anaerobic conditions for preparing solvents and setting up the reactors.

Furthermore, the detection of hydrogen peroxide in the liquors from DA/Fe ion pretreatments of biomass suggests a view of DA/Fe ion pretreatments as a thermal Fenton reaction where cycling between Fe^2+^ and Fe^3+^ allows the Fenton reaction to repeat, generating hydroxyl radicals that can aid in biomass deconstruction.

## Figures and Tables

**Table 1:** Release of glucose and xylose from corn stover by DA/Fe pretreatments (at 150 °C, 20 min) under anaerobic conditions. The released sugars were quantitated by high-performance liquid chromatography (HPLC) analysis of the liquor after the pretreatment. Values are presented as means ± standard error of the mean (± SEM) of four replicates for each of the pretreatments. Asterisk indicates statistically significant difference from the control (* for *p* < 0.05; ** for *p* < 0.01). Abbreviations: Ctrl, control; DA, dilute acid; Glu, glucose; NA, not applicable; Xyl, xylose.

Pretreatments (Anaerobic)	Glu Release	Increase	Xyl Release	Increase
	g per 100 g CS	% over Ctrl	g per 100 g CS	% over Ctrl
DA alone (Ctrl)	3.17 ± 0.06	NA	16.81 ± 0.47	-
DA/5 mM Fe^2+^	3.58 ± 0.01	17.1% *	18.94 ± 0.27	16.5% *
DA/10 mM Fe^2+^	3.75 ± 0.04	23.8% **	19.96 ± 0.26	24.4% **
DA/5 mM Fe^3+^	3.56 ± 0.09	15.9% *	18.88 ± 0.45	16.1% *
DA/10 mM Fe^3+^	3.68 ± 0.04	21.0% **	19.25 ± 0.37	19.0% **

**Table 2:** Release of glucose and xylose from corn stover by DA/Fe pretreatments (at 150 °C, 20 min) under aerobic conditions. The released sugars were quantitated by HPLC analysis of the liquor after the pretreatment. Values are presented as means ± standard error of the mean (± SEM) of four replicates for each of the pretreatments. Asterisk indicates statistically significant difference from the control (* for *p* < 0.05; ** for *p* < 0.01). Abbreviations: CS, corn stover; Ctrl, control; DA, dilute acid; Glu, glucose; NA, not applicable; Xyl, xylose.

Pretreatments (Aerobic)	Glu Release	Increase	Xyl Release	Increase
	g per 100 g CS	% over Ctrl	g per 100 g CS	% over Ctrl
DA alone (Ctrl)	3.03 ± 0.08	NA	14.87 ± 0.73	NA
DA/5 mM Fe^2+^	3.24 ± 0.05	7.1%	16.24 ± 0.11	9.2% *
DA/10 mM Fe^2+^	3.56 ± 0.03	17.4% *	17.67 ± 0.24	18.8% **
DA/5 mM Fe^3+^	3.21 ± 0.05	6.0%	16.00 ± 0.10	7.6%
DA/10 mM Fe^3+^	3.50 ± 0.05	15.6% *	17.01 ± 0.49	14.4% *

**Table 3:** Pretreatment conditions specifying the quantity of biomass, iron co-catalyst and solvent for dilute acid pretreatments. Five replicates were conducted for each of the treatments. Abbreviation: ddH_2_O, deionized distilled water.

Pretreatments	Description of Components
**Small scale DA/Fe pretreatment in 2-mL glass vials**	
DA alone (Ctrl)	100 mg CS + 1 mL 0.5% H_2_SO_4_
DA/5 mM Fe^2+^	100 mg CS + 1 mL 5 mM FeCl_2_ in 0.5% H_2_SO_4_
DA/10 mM Fe^2+^	100 mg CS + 1 mL 10 mM FeCl_2_ in 0.5% H_2_SO_4_
DA/5 mM Fe^3+^	100 mg CS + 1 mL 5 mM FeCl_3_ in 0.5% H_2_SO_4_
DA/10 mM Fe^3+^	100 mg CS + 1 mL 10 mM FeCl_3_ in 0.5% H_2_SO_4_
**Intermediate scale DA/Fe pretreatment in 65-mL pressure tubes**	
DA alone (Ctrl)	3 g CS + 30 mL 0.5% H_2_SO_4_
DA/10 mM Fe^2+^	3 g CS + 30 mL 10 mM FeCl_2_ in 0.5% H_2_SO_4_
DA/10 mM Fe^3+^	3 g CS + 30 mL 10 mM FeCl_3_ in 0.5% H_2_SO_4_

Notes: Corn stover (Pioneer variety 33A14) was harvested, and ground at the Kramer farm located in Wray, Colorado, and was further milled at NREL using a Wiley Mill to pass through a 20-mesh screen for 1-mm particle size.

## Abbreviations

CS	corn stover
DA	dilute acid
